# Effects of thermophilic and acidophilic microbial consortia on maize wet-milling steeping

**DOI:** 10.1186/s40643-024-00783-3

**Published:** 2024-07-16

**Authors:** Yaqin Sun, Wenjing Xia, Langjun Tang, Zhilong Xiu, Weiwu Jin, Xiaoyan Wang, Jin Tao, Haijun Liu, Hongyan An, Yi Li, Yi Tong

**Affiliations:** 1 MOE Key Laboratory of Bio-Intelligent Manufacturing, School of Bioengineering, Dalian University of Technology, No. 2 Linggong Road, Ganjingzi District, Dalian City, Liaoning Province 116024 P.R. China; 2 Jilin COFCO Biochemistry Co., Ltd. (National Engineering Research Center of Corn Deep Processing), 1717 Xiantai Street, Nanguan District, Changchun City, Jilin Province 130033 P.R. China

**Keywords:** Maize steeping, Microbial consortium, Thermophilic and acidophilic, Protein matrix, Adaptive evolution engineering

## Abstract

To understand the ecology of species and promote biotechnology through beneficial strain selection for improving starch yield in maize wet-milling steeping, bacterial diversity and community structure during the counter-current steeping process in a commercial steeping system were characterized and investigated. The microbial diversity in the steeping liquor, which consisted of 16 phyla, 131 families, and 290 genera, was more abundant compared to those present on the surface of unsteeped maize. As the counter-current steeping progressed, exposing newer maize to the older steepwater, *Lactobacillus* dominated, replacing *Rahnella*, *Pseudomonas*, *Pantoea*, and *Serratia*. The thermophilic and acidophilic microbial consortia were enriched through adaptive evolution engineering and employed to improve starch yield. Several steeping strategies were evaluated, including water alone, SO_2_ alone, mono-culture of *B. coagulans*, microbial consortia, and a combination of consortium and SO_2_. Combining the microbial consortium with SO_2_ significantly increased the starch yield to, about 66.4 ± 0.5%, a 22% and 46% increase over SO_2_ alone and the consortium alone, respectively. Scanning electron microscope (SEM) of steeped maize structure indicated that the combination of consortium and SO_2_ disrupted the protein matrix and widened gaps between starch granules in maize endosperm. This released proteins into the steepwater and left starch granules in the aleurone layer. The steeping strategy of using thermophilic and acidophilic microbial consortium as additives shows potential application as an environmentally friendly alternative to conventional maize steeping procedures.

**Supplementary Information:**

The online version contains supplementary material available at 10.1186/s40643-024-00783-3.

## Highlights

Characterization of bacterial diversity in a commercial steeping system.

Adaptation of a thermophilic and acidophilic microbial consortium for maize steeping.

Development of an environmentally friendly strategy for maize steeping.

Consortium combining SO_2_ leads to 66.4% increase in starch yield.

Novel strategy disrupts protein matrix making starch granules to aleurone layer.

**Supplementary Information:**

The online version contains supplementary material available at 10.1186/s40643-024-00783-3.

## Introduction

Wet-milling is the process of steeping maize and chemically separating and removing starch, protein (gluten), oil, and fiber from the maize kernel(Paulsen et al. 2003). Steeping is critical for preparing the kernels for milling and commonly follows a counter-current manner in industrial settings. At Jilin COFCO Biochemistry Co., Ltd (China), there is a battery of 12 conical-bottomed tanks with a capacity of 0.8 million tons per year. The newest maize is exposed to the oldest steepwater, while the oldest maize is contacted with the newest steepwater. Fresh steepwater, to which 0.10–0.20% (v/v) sulfur dioxide has been added, is continuously applied to the steep tank containing the oldest steeping corn. Sulfur dioxide, either in liquid form or produced by burning sulfur onsite, disrupts disulfide bonds in the endosperm protein matrix and limits bacterial growth. During steeping, the pH is maintained within the range of 3.6–4.8, and the steeping temperature is controlled at 50 °C.

As sulfur dioxide is one of the six most common air pollutants, research has explored alternatives like lactic acid, microorganisms, and enzyme to enhance steeping efficiency, decrease SO_2_ usage, and increase starch yield (Dailey et al. 2000; Ramírez et al. 2009). Lactic acid (LA) in the steeping process is known to improve wet-milling starch yields as it helps break down the endosperm protein matrix (Singh et al. 1997, 1999; Dailey et al. 2000). When 0.55% LA was added into the steepwater, starch yields increased by 3–12% for 18 hard and soft dent varieties (Singh et al. 1997). The effect of LA, SO_2_, and a combination of LA and SO_2_ on the solubilization of protein was investigated (Dailey et al. 2000). The initial slow rate of protein solubilization appeared to be due to incomplete kernel hydration. Additionally, the effect of steeping additives on the quality of isolated tef starch was investigated (Nyakabau et al. 2013). The results showed that a combination of SO_2_ and lactic acid improved the starch yield, but steeping with sodium hydroxide produced highest starch purity. The presence of LA resulted in significantly greater amounts of released protein compared to its absence, with the highest amounts found when steeping was performed with both LA and SO_2_. The proteinaceous material in industrial steepwater was reported to originate from the maize itself rather than from microbial fermentation, as indicated by the similarity in amino acid distributions between the steepwater and maize protein fractions (Hull et al. 1996a, b). Enzymatic wet milling (E-milling) was developed as an environmentally friendly alternative for traditional maize wet-milling, eliminating the need for sulfites (Johnston and Singh 2001). An E-milling model was presented to estimate the production cost per kilogram of starch, and the results indicated that the E-milling process was cost-competitive with the conventional process during periods of high maize feedstock costs (Ramírez et al. 2009).

Maize steeping is a complex physical, chemical, and biological process, and the microbial consortia participating in the maize steeping process play an important role that should not be ignored. Microbial consortia are extensively employed in traditional food fermentation and large-scale chemicals and high-value product manufacturing due to their ability to withstand environmental fluctuations and perform complex tasks effectively (Hanemaaijer et al. 2015; Qian et al. 2020; Sgobba and Wendisch 2020). Moreover, microbial consortia are applied in lactic acid production, offering advantages such as high adaptability to raw materials, simple nutrient requirements, and unaffected performance under non-sterile conditions (Sun et al. 2019, 2021). However, despite the previously mentioned reports, there needs to be more information regarding microbial diversity and communities interacting during counter-current steeping. This study aims to characterize and investigate the microbial communities associated with maize steeping during the counter-current steeping process in a commercial steeping system. Additionally, this study aims to discover and adapt microbial communities from the maize steeping to improve starch yield, reduce the amount of SO_2_, and shorten the steeping time. The structures of maize under different steeping strategies were compared to establish the relationship between the microbial consortium and steeping.

## Materials and methods

### Materials and medium

The maize used in this study was grown during the 2018–2020 crop season in Jilin and Heilongjiang province of China. Upon arrival at the laboratory, the maize was stored in a refrigerator at 4 °C. All other chemicals were of reagent grade and commercially available.

The enrichment medium was as follows: 20 g/L glucose, 10 g/L beef extract, 10 g/L peptone, 5 g/L yeast extract, 2 g/L ammonium citrate, 5 g/L sodium acetate, 2 g/L K_2_HPO_4_, 0.20 g/L MgSO_4_, 0.05 g/L MnSO_4_·H_2_O.

The fermentation medium (CSLP medium) used was as follows: 20 g/L glucose, 16 g/L corn steep liquor power (CSLP), 2 g/L ammonium citrate, 2 g/L sodium acetate, 2 g/L K_2_HPO_4_, 0.2 g/L MgSO_4_·7H_2_O, and 0.05 g/L MnSO_4_·H_2_O.

### Adaptive evolution engineering of the microbial consortium to temperature and pH

The adaptation protocol was established to obtain the thermophilic and acidophilic microbial consortia applied in the steeping process. Maize steeping liquor samples (2.5 mL) were first inoculated into the enrichment medium (50 mL). The enrichment broth was transferred to a CSLP medium (100 mL) with 1% (v/v) incubation using an adaptive strategy of decreasing pH (5.0, 4.6, 4.4, 4.0) at 50 °C. The culture was cultivated for 48 h and then transferred to a fresh adaptation medium with a lower pH. The culture was cultivated five times at the same pH before exposure to the lower one. After 20 generations of long-term domestication, the stable thermophilic and acidophilic microbial consortium DUT21 was achieved.

### The construction of diluted consortia and isolation of single strain

Mini consortia were constructed by serial dilution (10^− 2^ to 10^− 8^) of the original consortium DUT21 with sterile saline and then incubated in the CSLP medium. Once sugars were depleted, the enriched consortia were serially transferred to the fresh seed medium with a 1% (v/v) inoculation three times to ensure a stable microbial composition. By streaking on a solid medium with bromocresol green as an indicator, single strains from consortium DUT21 were isolated and purified. As lactic acid production increased, the indicator’s color gradually shifted from blue to green. Two strains with perfect performance were chosen. BLAST analysis of the 16 S rRNA gene sequence of the isolated two strains demonstrated 100% similarity to *Bacillus coagulans*. The 16 S rRNA sequences of *B. coagulans* S1 and S2 were submitted to the GenBank database with accession numbers OK655897 and OK655939, respectively. The *B. coagulans* S1 strain has already been deposited at the China General Microorganism Collection Center (CGMCC No. 23,993).

### Composition analysis of the microbial consortium

The bacterial community compositions of microbial consortia during the steeping process, consortium DUT21, and its dilution consortia were investigated by 16 S rRNA gene amplicon high-throughput sequencing provided by Sangon Biotech in Shanghai, China. 16 S rRNA gene sequences for the consortium DUT21 and its dilution consortia have been submitted to the NCBI Sequence Read Archive, and the corresponding accession numbers and composition are shown in Table 1.

**Table 1:** Microbial community analysis of the adaptive consortium DUT21 and its diluted consortia

Taxonomy	Percentage (%)				
	DUT21 *SRR16685566	Diluted consortia of DUT21			
		DUT21-2 ( ×10^− 2^ ) *SRR16685586	DUT21-4 ( ×10^− 4^ ) *SRR16685583	DUT21-6 ( ×10^− 6^ ) *SRR16685585	DUT21-8 ( ×10^− 8^ ) *SRR16685584
*Bacillus*	99.48	99.46	99.50	99.62	99.56
*Lactococcus*	0.01	0.01	0.02	0.01	0.01
*Serratia*	0.01	0.02	0.01	0.02	0.02
*Chryseomicrobiu*	0.06	0.03	0.03	0.02	0.04
*Exiguobacterium*	0.01	0.01	0.01	0.01	0.01
*Bellilinea*	0.01	-	-	0.01	0.01
*Empedobacter*	-	-	0.02	0.01	0.01
*Gluconacetobacter*	0.02	0.02	0.01	-	0.02
*Streptophyta*	0.03	0.02	0.02	0.02	0.01
*Thauera*	0.01	0.01	0.02	0.01	0.01
Unclassified	0.37	0.44	0.37	0.29	0.31

*The accession number of microbial consortia in NCBI Sequence Read Archive

### Laboratory wet-milling

The laboratory wet-milling procedure was performed under a non-sterilized condition in a 250 mL serum bottle filled with 150 mL water and 40 g maize. Steeping was investigated for solutions containing water, SO_2_ (600–1000 ppm), mono-culture of *B. coagulans*, microbial consortia, and a combination of consortia and SO_2_. The steeping was agitated at 200 rpm and 50 °C for the designated steeping time.

Immediately after steeping, the mass of the steeped maize was measured with water removal from the surface of the maize. Starch was extracted following the laboratory wet-milling procedure described previously (Pérez et al. 2001). Some details were improved. Steeped maize was ground in 100 mL of distilled water using a grinder. The water slurry was manually sieved through a set of stainless steel screens of 40-mesh and 200-mesh. Germ and fiber were retained in the first screen, and protein in the second. The starch slurry was passed through 200-mesh and stood at 4 °C overnight. The starch slurry was then centrifuged at 3700 rpm for 20 min to remove protein further. The starch was collected and dried to a constant weight and then weighed. The maize starch yield (Y) was calculated by dividing the dry matter of starch weight by the dry matter of steeped maize, expressed in g/g.

### Analytical methods

SO_2_ concentration was determined by the iodometric titration method. Steepwater of 5 mL was added to distilled water of 20 mL and starch indicator of 1 mL. The mixture was mixed and shaken well. The mixture was dripped with standard iodine solution (0.05 mol/L) until blue, and the volume of iodine solution consumed was recorded.

For each sample, the protein concentration in the steepwater was analyzed using the Kjeldahl method. The moisture content of the steeped maize was determined by drying three 10-g maize samples in the oven at 105 °C to constant weight.

Glucose, lactic acid, critic acid, and acetic acid were analyzed using high-performance liquid chromatography (HPLC) equipped with an Aminex HPX-87 H column with a column temperature of 65 °C. Sulfuric acid (5 mmol/L) was the mobile phase with a 0.6 mL/min flow rate.

### Statistical analyses

All steeping experiments were conducted in triplicate. Standard deviations and coefficients of variance were calculated, and the data were subjected to IBM SPSS analysis of variance (ANOVA) procedures to test for significant differences among the steep conditions. A p-value < 0.05 was taken as an indication of a significant difference.

## Results and discussion

### Community structures of microbial consortia in a commercial maize wet-milling steeping system

**Fig. 1:**
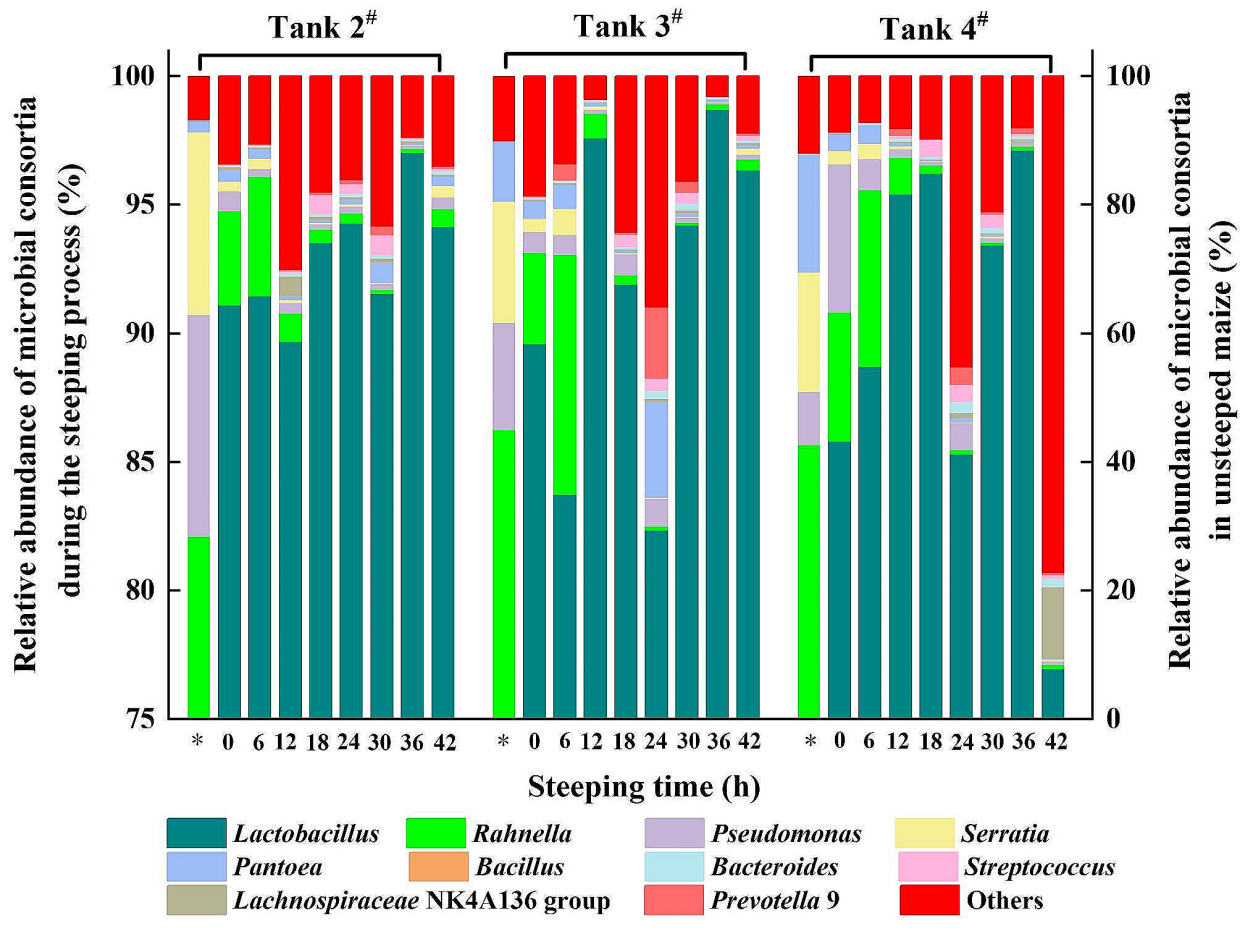
Relative abundance of microbial consortia in unsteeped maize and during the steeping process. Samples were taken from a commercial maize steeping system in Jilin COFCO Biochemistry Co., Ltd (China) including a battery of 12 conical-bottomed tanks of 0.8 million tons capacity per year. Each tank has a volume of 668 m^3^, with a loading capacity of 90% for maize (v/v). The fresh steepwater sequentially passes through tank 4^#^, tank 3^#^, and tank 2^#^. The steeping temperature for tanks 2^#^-4^#^ are 49.8, 50.4, and 50.5 ^o^C, respectively

In this study, the influence of microbial community on the countercurrent steeping process in commercial steeping systems at Jilin COFCO Biochemistry Co., Ltd (China) was investigated. The steeping systems consisted of a battery of 12 conical-bottomed tanks, each with a capacity of 668 m^3^. 16 S rRNA gene amplicon high-throughput sequencing was performed to investigate the bacterial composition of microbial consortia in steeping tanks 2^#^, 3^#^, and 4^#^ during steeping. Figure 1 illustrates the evolution of microbial community structure throughout the steeping process. On the surface of unsteeped maize, 416 OTUs were identified, encompassing 9 phyla, 66 families, and 151 genera. The steeping liquor exhibited a higher microbial diversity, with 16 phyla, 131 families, and 290 genera. The bacterial composition on the maize surface varied due to the different origins of the maize. Tanks 2^#^ and 3^#^ had similar dominant families: *Rahnella*, *Pseudomonas*, and *Serratia*, representing 90.97% and 80.41% of total abundance, respectively. However, in steeping tank 4^#^, *Pantoea*, with an abundance of 18.25%, replaced *Pseudomonas* (8.28%) as one of the dominant families. The analysis of the 16 S rRNA gene sequencing from the oldest steepwater revealed that *Lactobacillus* was the dominant family, constituting approximately 90% of the total abundance. As the newest maize was exposed to the oldest steepwater in the countercurrent steeping process, *Lactobacillus* became the primary family in the investigated tanks, replacing *Rahnella*, *Pseudomonas*, *Pantoea*, and *Serratia*. Throughout the maize steeping process, *Lactobacillus* consistently remained the dominant family, reaching an abundance of over 95% after 36 h. However, the abundance of *Lactobacillus* in tank 4^#^ decreased to 77% at 42 h, possibly due to presence of other unanalyzed genera. Additionally, the genera *Bacillus*, *Bacteroides*, *Streptococcus*, and *Prevotella*9 occupied less than 5% abundance in the steeping liquor. These diverse bacteria working together led to a starch yield exceeding 69% throughout the countercurrent steeping process at the factory.

**Fig. 2:**
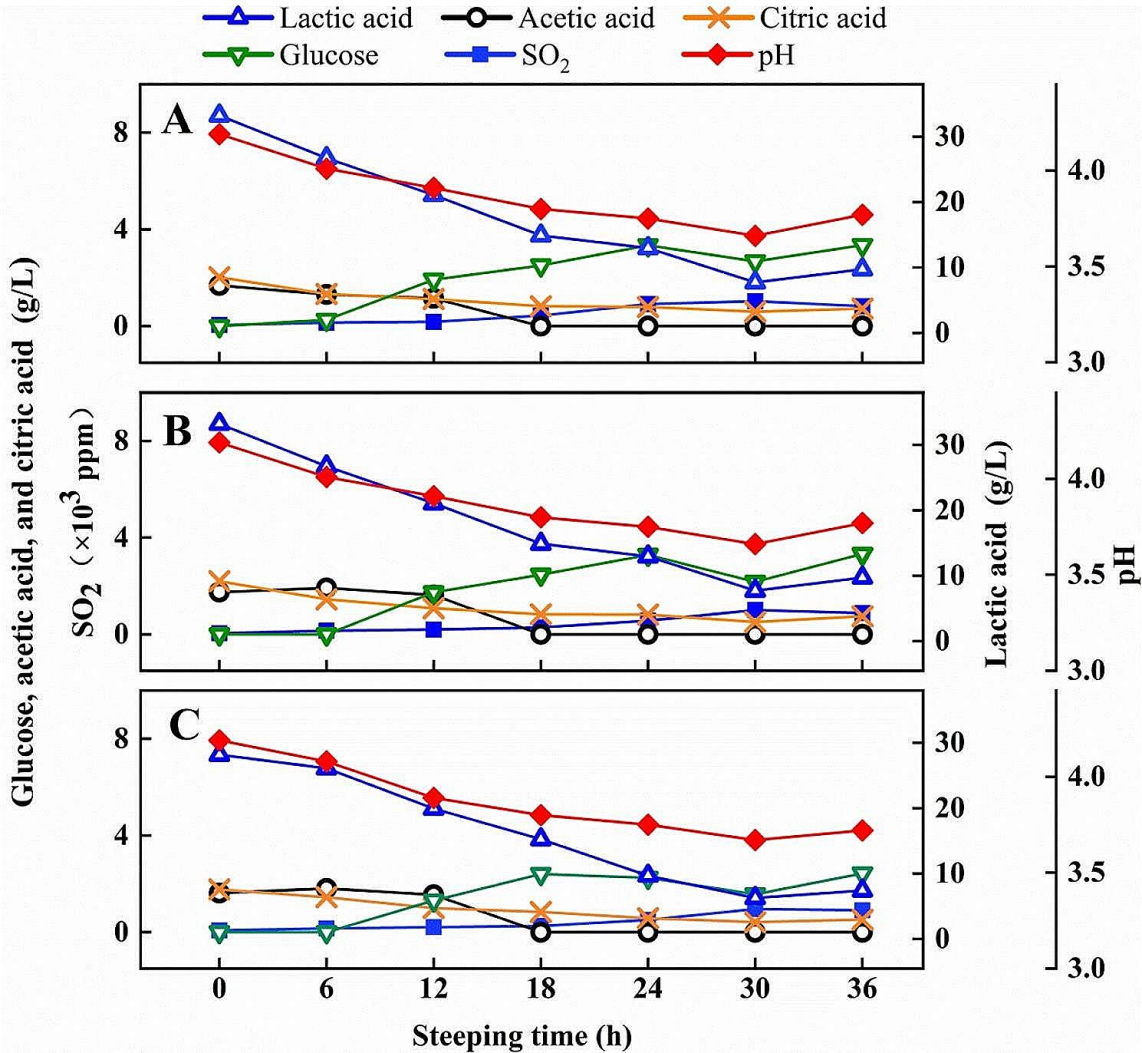
The composition analysis of the steeping liquor during the steeping process, **(A)** Tank 2^#^; **(B)** Tank 3^#^; **(C)** Tank 4^#^. Samples were taken from a commercial maize steeping system in Jilin COFCO Biochemistry Co., Ltd (China) including a battery of 12 conical-bottomed tanks of 0.8 million tons capacity per year

### The composition analysis of the steeping liquor in a commercial wet-milling steeping system

As steepwater circulates, carbohydrates and sulfur dioxide concentration increase. Conversely, lactic acid, acetic acid, critic acid, and succinic acid concentrations decline. Lactic acid decreased from around 30 g/L to 7 g/L after 36 h of steeping (See Fig. 2). Acetic acid, critic acid, and succinic acid remain below 3 g/L. Despite the decrease in organic acids, the pH of steeping liquor decreases due to the increased SO_2_ concentration, resulting within a range of 3.6 to 4.2. Studies indicate that starch yields decline when initial pH values exceed 4.0, with no noticeable impact when pH is lower (Cabrales et al. 2006). At the initial steeping stage, SO_2_ is maintained at a low concentration (< 100 ppm), but with extended steeping time, the maximum SO_2_ concentration can reach over 1000 ppm. SO_2_ has been shown to facilitate starch particle release from the endosperm protein matrix (Mitsuo and ShokoI, 1982). Moreover, the effects of various acids, including lactic acid, acetic acid, hydrochloric acid, oxalic acid, phosphoric acid, and sulfuric acid, on wet-milling yields and starch properties have been studied (Yang et al. 2005). Weak acids, such as lactic acid and acetic acid, generally lead to higher starch yields compared to strong acids (such as hydrochloric acid, oxalic acid, phosphoric acid, and sulfuric acid). No apparent trends were observed in the effects of different acids on starch pasting properties. Additionally, the effect of lactic acid addition on starch yield was tested on 18 commercial corn hybrids, and the magnitude of the increased starch yields varied between 2.9 and 12.0% (Singh et al. 1997).

### Adaptive evolution engineering of the thermophilic and acidophilic microbial consortia

In this study, a thermophilic and acidophilic microbial consortium was enriched and adapted using an adaptive evolution strategy. The pH was gradually decreased from 5.0 to 4.0 while maintain a high temperature of 50 °C. The long-term domestication resulted in a stable and functional microbial consortium (termed DUT21) with dual tolerance to the temperature and acidity. The bacterial composition of the obtained DUT21 consortium was investigated using high-throughput sequencing of the 16 S rRNA gene amplicon, and the results are presented in Table 1. The predominant genus was *Bacillus*, accounting for 99.48% of the total microorganism relative abundance. In an attempt to eliminate other genera, the DUT21 consortium was diluted, and the abundance of diluted consortia was also evaluated (Table 1). It was challenging to completely eliminate the other genera through dilution, and even after 10^− 8^ dilutions, approximately 0.44% abundance of other genera remained.

Isolating and purifying individual strains from the DUT21 consortium were carried out. BLAST analysis of the 16 S rRNA gene sequence of the isolated strain demonstrated 100% similarity to *Bacillus coagulans* ATCC 7050. The strain was then conserved at the China General Microbiological Culture Collection Center (CGMCC) with the preservation number CGMCC 23,993.

### Effect of thermophilic and acidophilic microbial consortia on starch yield

**Fig. 3:**
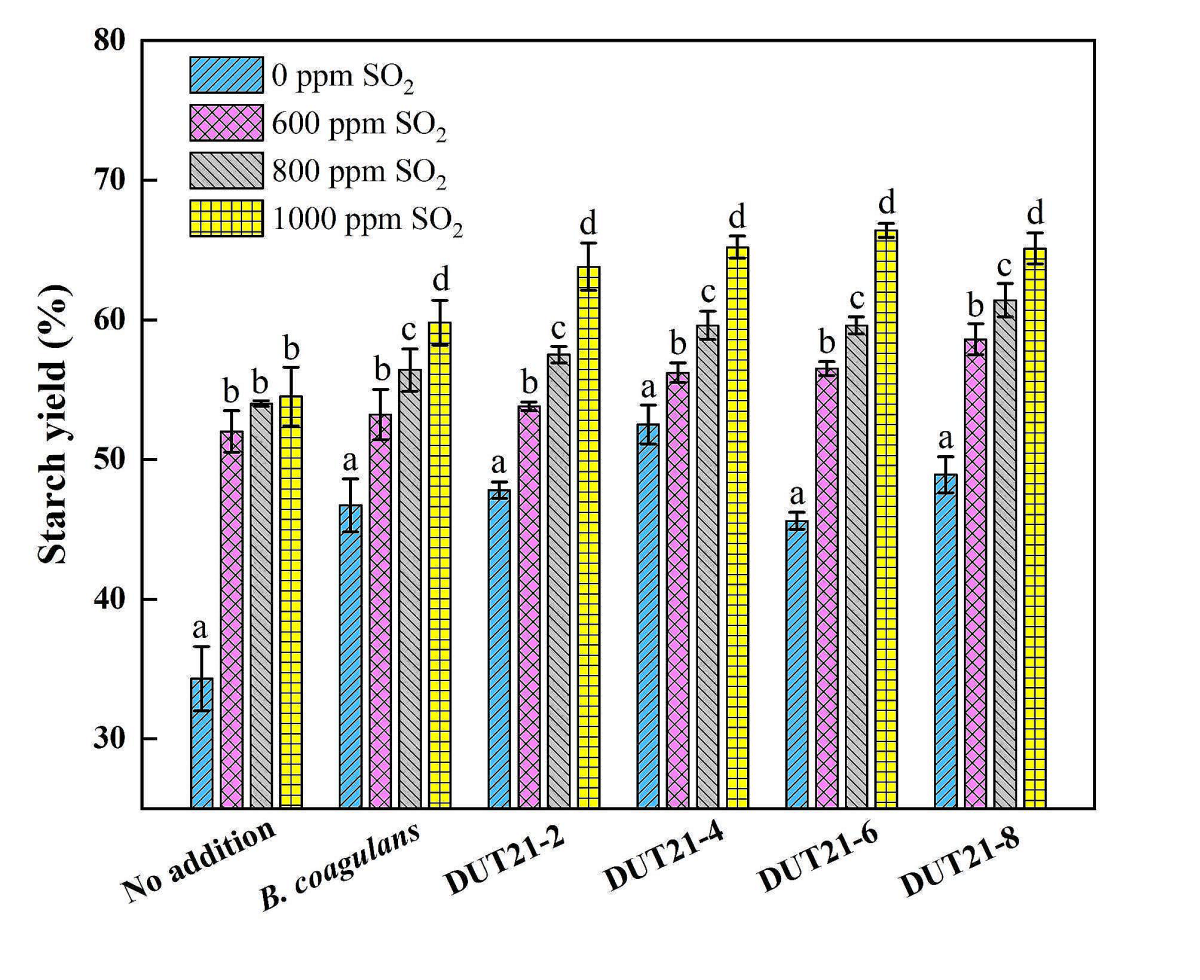
Effect of microbial consortia, *B. coagulans*, and SO_2_ concentration on starch yield. Steeping was carried out in a 250 mL serum bottle containing 40 g maize and 150 mL water at 50 ^o^C and 200 rpm for 44 h. The inoculation size of microbial consortia and *B. coagulans* was 10% (v/v)

The performance of diluted consortia and *B. coagulans* CGMCC 23,993 on improving starch yield in steeped maize was investigated and evaluated (Fig. 3). All microbial consortia and *B. coagulans* demonstrated excellent performance in enhancing starch yield compared to treatments without any consortium addition, particularly when varying SO_2_ concentrations from 0 to 1000 ppm. Starch yield increased with higher bisulfite concentration, which is consistent with previous reports (Singh et al. 1999; Yang et al. 1999). Bisulfite contributes to higher starch yields by disrupting protein matrixes through breaking disulfide bonds.

The starch yields for steeped maize in the investigated steeping solutions ranged from approximately 34.3 ± 2.3% to 66.4 ± 0.5%. Adding the investigated dilute microbial consortia and *B. coagulans* significantly enhanced starch yield compared to the control group, which had yields of approximately roughly 33-53% without SO_2_ supplementation. As the SO_2_ concentration increased, the improvement in starch yield became less pronounced. Notably, a combination of microbial consortium DUT21-6 (10% (v/v) inoculation) and SO_2_ (1000 ppm) resulted in a substantial increase in starch yield to approximately 66.4 ± 0.5%. This represented a 22% increase over SO_2_ alone and a 46% increase compared to microbial consortium alone. The effect of the inoculation sizes of the microbial consortium on starch yield was also evaluated, revealing little statistical difference. As the inoculation proportion increased from 8% (v/v) to 12% (v/v), starch yields varied approximately 62.0 ± 1.1% to 65.4 ± 3.0%.

The effect of mono-culture of *B. coagulans* CGMCC 23,993 on starch yield showed a gradual increase from 53.2 ± 1.8% to 59.8 ± 1.6% as the SO_2_ concentration varied from 600 ppm to 1000 ppm. These results indicate that microbial consortia have a positive impact on starch yield during the steeping process. This improvement may be attributed to the combination of different *B. coagulans* or other genera present in very small amounts within the consortia.

Previous report have investigated the effects of various methods, including adding lactic acid (Singh et al. 1999), SO_2_(Singh et al. 1999; Yang et al. 1999), mono-culture of *Lactobacillus* (Hull et al. 1996a), enzyme (Ramírez et al. 2009; Sharma and Tejinder 2014), and chemical pretreatments using KOH and ethyl oleate (Haros and Suárez 1999), either individually or in combination (Dailey 2002). The highest starch yield of 76.1 ± 0.3% was achieved through pretreatment with KOH (1%), followed by steeping with lactic acid (0.5%, v/v) and SO_2_ (0.25%) (Haros and Suárez 1999).

### Effect of different steeping strategies on steepwater protein and moisture contents of steeped maize

**Fig. 4:**
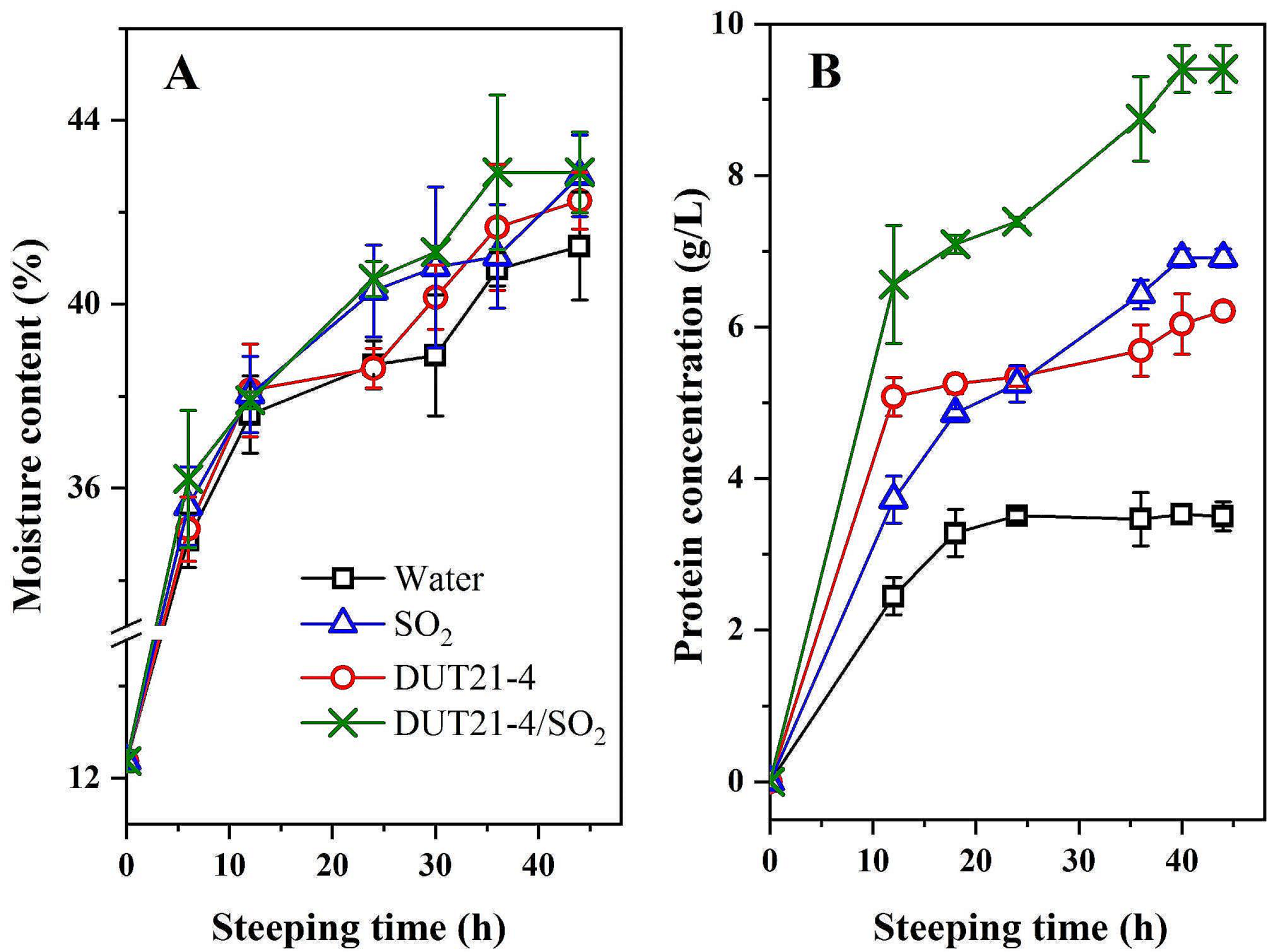
Effect of different steeping strategies on moisture content **(A)** and protein solubilization **(B)**. Steeping was carried out in a 250 mL serum bottle containing 40 g maize and 150 mL water at 50 °C and 200 rpm. SO_2_ concentration of 1000 ppm and 10% (v/v) inoculation of consortium DUT21-4 were selected

In this study, steepwater protein and moisture content were evaluated for four different steeping strategies: distilled water (control), SO_2_, consortium DUT21-4, and a combination of DUT21-4 and SO_2_ as steeping additives at 50 °C with steep times of up to 44 h (shown in Fig. 4). The initial moisture content of the maize was measured at 12.37 ± 0.23%. During the initial 6 h steeping period, there was rapid water uptake, after which the rate slowed down. Moisture equilibrated was reached at around 12 h, and different steeping strategies did not have a significant impact on moisture content. Regardless of the steeping strategy employed, the final moisture content stabilized at approximately 41-42%. Similar moisture content has been reported when using SO_2_ or/and lactic acid as steeping additives (Dailey et al. 2000; Sharma and Tejinder 2014). Additionally, studies have indicated that alkaline pretreatment with KOH solutions can enhance water uptake, possibly due to the alkali’s effect on the maize’s pericarp (Haros and Suárez 1999).

Protein concentration in the steepwater increased with the duration of steeping for all the steeping strategies. Significantly higher amounts of protein were released in the presence of SO_2_ and microbial consortium compared to when they were absent. The protein solubilization induced by microbial consortium DUT21-4 showed similar trends to that induced by SO_2_. As a result, the combination of SO_2_ and consortium DUT21-4 resulted in the highest protein concentrations compared to the individual water, SO_2_, or consortium DUT21-4 treatments (Fig. 4B). The protein concentration followed the order of SO_2_/DUT21-4 (9.41 ± 0.31 g/L) > SO_2_ (6.91 ± 0.12 g/L) > DUT21-4 (6.21 ± 0.13 g/L) > water (3.5 ± 0.19 g/L). Another study reported a protein yield of about 9.5 g/L when steeping was treated with a combination of lactic acid (0.50%) and SO_2_ (2000 ppm) at 52 °C (Dailey et al. 2000). The solubilization of kernel protein with lactic acid was found to be very sensitive to temperature compared to the protein release induced by SO_2_. Additionally, a significant drop in steepwater protein between 20 and 40 h was observed during the SO_2_ steeping process (Hull et al. 1996a). They also reported a higher protein concentration with the addition of *Lactobacillus* (10.2 g/L) compared to steeping treated with lactic acid (5.5 g/L) and SO_2_ (3.1 g/L) at 40 h.

### Effect of different steeping strategies on maize structure

The major structural components of the maize consist of the pericarp, germ, endosperm, and tip cap (Jackson and Shandera 1995). Research indicates that the tip cap serves as the primary route for steepwater uptake into the kernel (Watson 1984). However, steepwater can also enter the tip cap, penetrate the open structure of the spongy cells that connect the tip cap to the pericarp, and flow through the loose, open layers between the pericarp and seed coat. The flux of steepwater is from the basal end of the kernel to the crown. The large intercellular spaces in the cross-cell layers provide a significant pathway for moisture transfer from the tip cap to the endosperm.

**Fig. 5:**
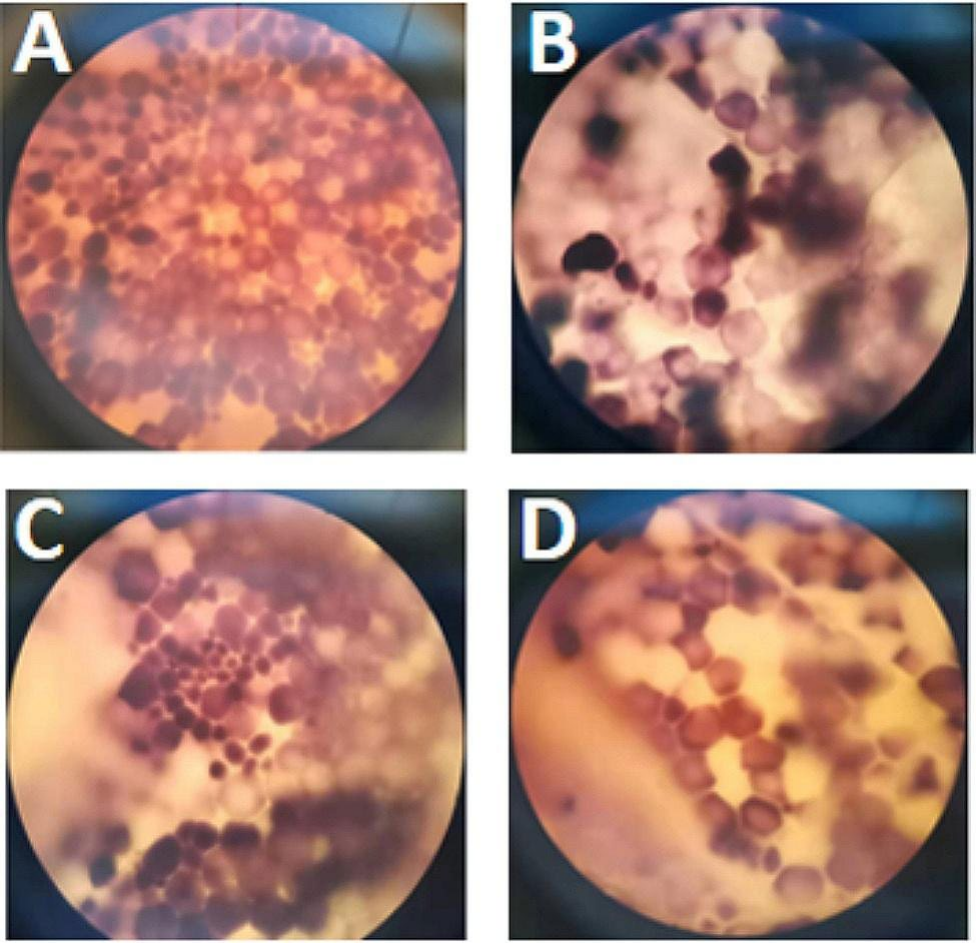
Effect of different steeping strategies on endosperm structure at a steeping time of 44 h under the light microscope. **(A)** Water alone; **(B)** SO_2_ alone, 1000 ppm; **(C)** Consortium DUT21-4 alone, 10% (v/v) inoculation; **(D)** SO_2_ (1000 ppm) and consortium DUT21-4 (10% (v/v) inoculation). All samples were observed at 100× magnification

In this study, the endosperm was investigated to observe the starch-protein matrix under various steeping strategies. After steeping, the endosperm samples were stained using the I-KI technique, and their structures were analyzed using an optical microscope (OM), as shown in Fig. 5. Starch granules appeared blue, while the proteins appeared yellow. It can be observed that the protein matrix in the endosperm tightly enveloped the starch particles after steeping in the water. However, the protein matrix structure in the endosperm region became partially separated from starch particles after steeping in consortium DUT21-4 (10%, v/v) and SO_2_ (1000 ppm). This separation was significantly apparent when both consortium DUT21-4 and SO_2_ were employed together in the steeping process.

**Fig. 6:**
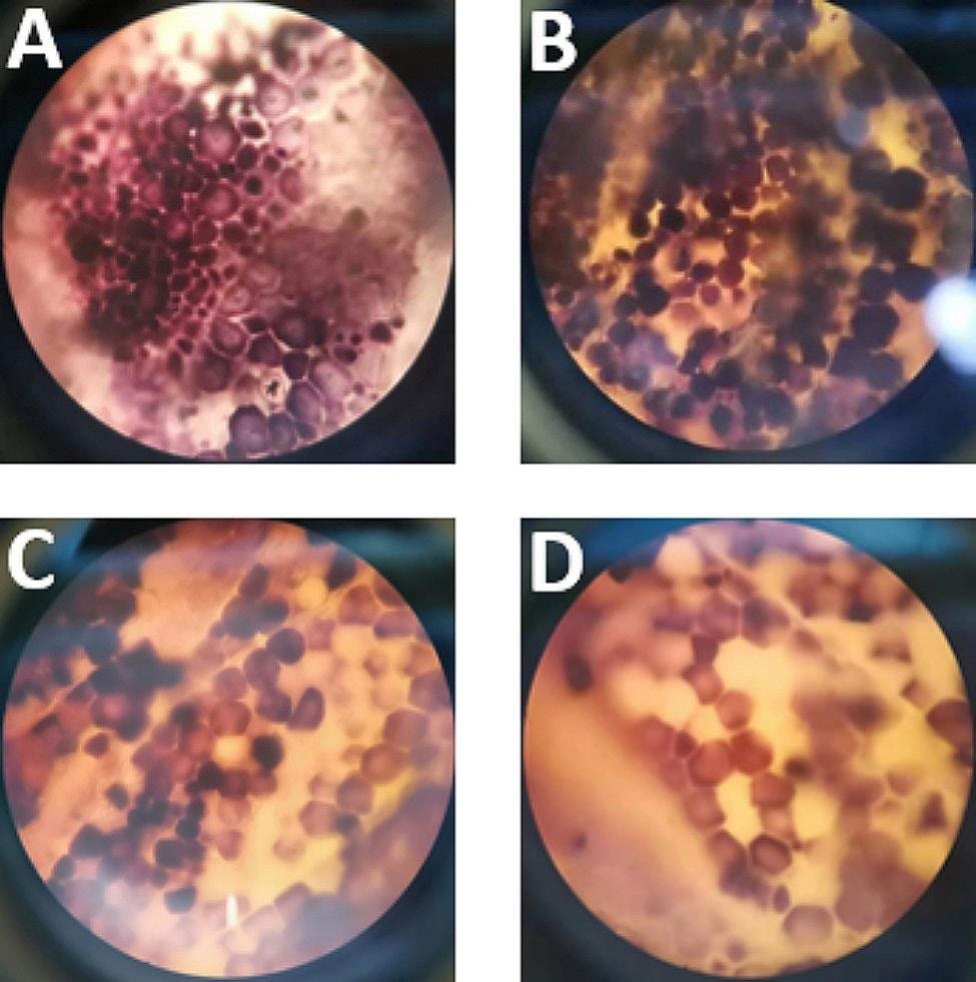
Effect of steeping time on endosperm structure under the light microscope during the steeping process of combination of SO_2_ and consortium DUT21-4. **(A)** 12 h; **(B)** 24 h; **(C)** 36 h; **(D)** 44 h. All samples were observed at 100× magnification

Additionally, the endosperm structures following staining were observed at varying steeping durations under the steeping combining consortium and SO_2_ (See Fig. 6). The protein matrix tightly wrapped starch granules after the first 12 and 24 h of soaking, but the structure of the protein matrix became loose after 36 h of soaking. These results indicate that the longer the soaking time, the looser the starch granules in the endosperm, making it easier to separate them. The combination of consortium and SO_2_ disrupted the protein matrix and further improved the transport of materials between the kernel and steepwater. The starch yield under different steeping times was also investigated, and the results indicated that the starch yield of 50.1 ± 1.5%, 61.4 ± 0.4%, 63.9 ± 0.4%, and 65.2 ± 0.8% at 24 h, 36 h, 40 h, and 44 h, respectively.

**Fig. 7:**
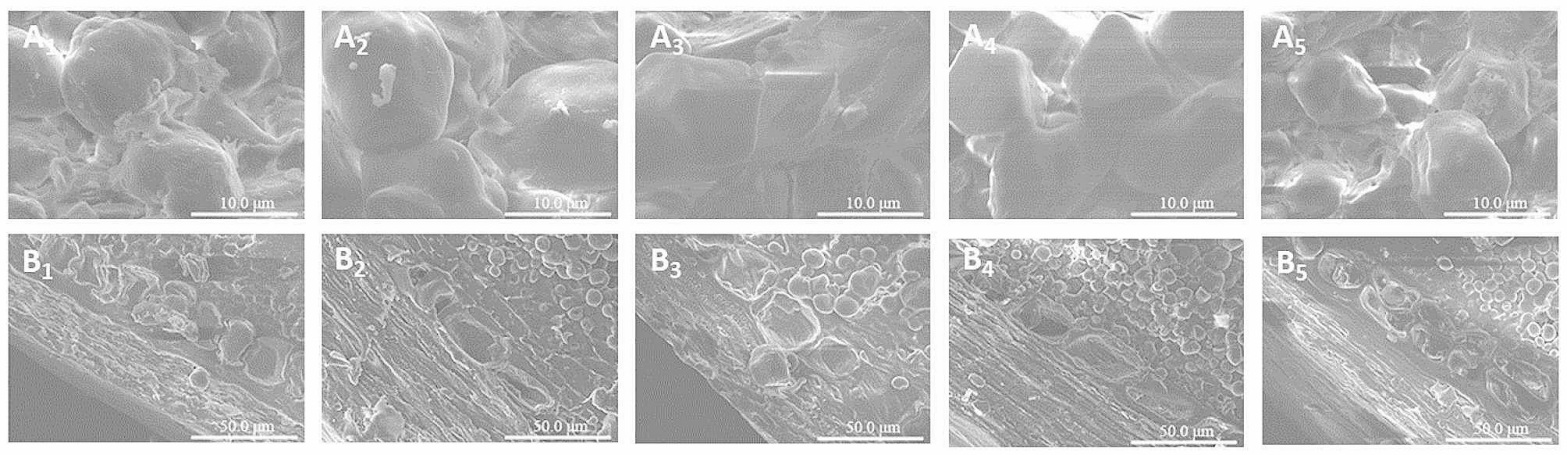
Scanning electron micrograph of the aleurone layer and endosperm of maize under different steeping strategies at a steeping time of 44 h. A and B represented endosperm and the aleurone layer, respectively. 1, 2, 3, 4, and 5 represented unsteeped maize, steeping with water alone, steeping with consortium DUT21-4 alone (10% (v/v) inoculation), steeping with SO_2_ alone (1000 ppm), and steeping with SO_2_ (1000 ppm) combing consortium DUT21-4 (10% (v/v) inoculation), respectively. The aleurone layer and endosperm were observed at 1000× and 5000× magnification, respectively

Furthermore, to analyze the changes in maize structure before and after steeping, the effect of different steep strategies on the aleurone layer and endosperm of maize were comprehensively observed using a scanning electron microscope (SEM). The aleurone layer, which is the outer layer of the endosperm, consists of a single layer of cells (See Fig. 7). These cells appear granular and contain protein but little or no starch (Watson 1984). Under the steeping strategy of combining the microbial consortium DUT21-4 with SO_2_, starch granules were observed in the aleurone layer. However, no starch granules were observed during the steeping strategies using water alone, SO_2_ alone, or consortium alone. These results imply that the bonding forces maintaining the matrix proteins together were more effectively weakened by the strategy of combining the microbial consortium with SO_2_. This blend led to the destruction of the protein matrix surrounding the starch granules in the maize endosperm and increased the gap between starch granules. Consequently, the proteins were released into the steepwater. Notably, some invisible holes were observed in the starch granules and protein matrix in the samples treated with both SO_2_ and the consortium.

## Conclusions

In this study, microbial diversity and composition within a commercial steeping system including a battery of 12 conical-bottomed tanks were investigated, aiming to clarify the influence of the microbial community on the countercurrent steeping process, The steeping liquor exhibited heightened biodiversity, consisted of 16 phyla, 131 families, and 290 genera, compared to the unsteeped maize. To improve starch yield and reduce SO_2_ utilization, adaptive evolution engineering was applied to enrich thermophilic and acidophilic microbial consortia as additives within the steeping liquor. Different steeping strategies, including water alone, SO_2_ alone, mono-culture of *B. coagulans*, microbial consortia, and a combination of consortium and SO_2_, were evaluated and compared. Our findings demonstrated that the combination of microbial consortium (10% (v/v) inoculation) and SO_2_ (1000 ppm) resulted in a substantial increase in starch yield to about 66.4 ± 0.5%, which was an increase of 22% and 46% compared to SO_2_ alone and microbial consortium alone, respectively. When applying the microbial consortium DUT21-4 combined with SO_2_, the protein matrix structure in the endosperm region was partially separated from starch granules, leading to significantly greater protein concentrations in steepwater compared to those treated with the water, SO_2_, or consortium. Starch granules appeared in the aleurone layer, indicating that the bonds holding the matrix proteins together were more loosely broken down. The combination of consortium and SO_2_ disrupted the protein matrix surrounding the starch granules and increased the gap between starch granules in maize endosperm, causing the protein to be released into the steepwater. The steeping strategy of using thermophilic and acidophilic microbial consortia as additives shows potential for industrial-scale application, as it can reduce SO_2_ consumption and improve starch yield.

### Electronic supplementary material

Below is the link to the electronic supplementary material.

Supplementary Material 1

## Data Availability

The datasets used and/or analyzed during the current study are available from the corresponding author on reasonable request.

## Abbreviations

SEM: Scanning electron microscope
LA: Lactic acid
E-milling: Enzymatic wet milling
CSLP: Corn steep liquor power
HPLC: High performance liquid chromatography
