# Supplementary figures and images for: Effects of thermophilic and acidophilic microbial consortia on maize wet-milling steeping
Source: Bioresour Bioprocess. 2024 Jul 16;11(1):68. doi: 10.1186/s40643-024-00783-3 (PMC11252109; doi:10.1186/s40643-024-00783-3)

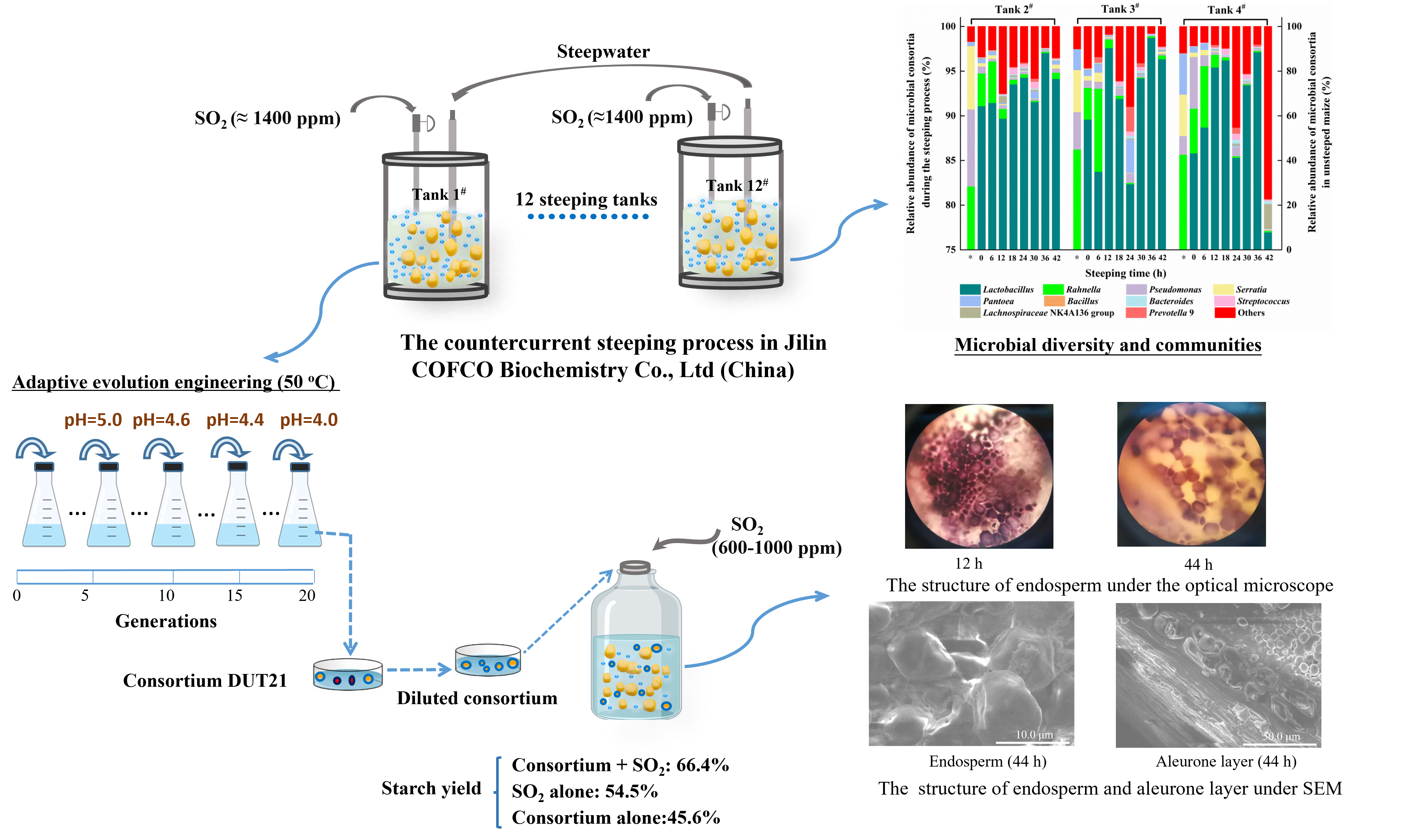

Supplement: Supplementary file 1 — Supplementary Material 1 [file 40643_2024_783_MOESM1_ESM.tif]
